# Arp2/3 Complex Is Required for Auxin-Driven Cell Expansion Through Regulation of Auxin Transporter Homeostasis

**DOI:** 10.3389/fpls.2020.00486

**Published:** 2020-04-28

**Authors:** Judith García-González, Štépánka Kebrlová, Matěj Semerák, Jozef Lacek, Innu Kotannal Baby, Jan Petrášek, Kateřina Schwarzerová

**Affiliations:** ^1^Department of Experimental Plant Biology, Faculty of Science, Charles University, Prague, Czechia; ^2^Institute of Experimental Botany, Czech Academy of Sciences, Prague, Czechia

**Keywords:** actin, cytoskeleton, auxin, cell expansion, Arp2/3 complex

## Abstract

The Arp2/3 complex is an actin nucleator shown to be required throughout plant morphogenesis, contributing to processes such as cell expansion, tissue differentiation or cell wall assembly. A recent publication demonstrated that plants lacking functional Arp2/3 complex also present defects in auxin distribution and transport. This work shows that Arp2/3 complex subunits are predominantly expressed in the provasculature, although other plant tissues also show promoter activity (e.g., cotyledons, apical meristems, or root tip). Moreover, auxin can trigger subunit expression, indicating a role of this phytohormone in mediating the complex activity. Further investigation of the functional interaction between Arp2/3 complex and auxin signaling also reveals their cooperation in determining pavement cell shape, presumably through the role of Arp2/3 complex in the correct auxin carrier trafficking. Young seedlings of *arpc5* mutants show increased auxin-triggered proteasomal degradation of DII-VENUS and altered PIN3 distribution, with higher levels of the protein in the vacuole. Closer observation of vacuolar morphology revealed the presence of a more fragmented vacuolar compartment when Arp2/3 function is abolished, hinting a generalized role of Arp2/3 complex in endomembrane function and protein trafficking.

## Introduction

The Arp2/3 complex is a conserved actin nucleator consisting of two actin-related proteins (ARP2 and ARP3) and five other complex-specific subunits (ARPC1 to ARPC5) (Welch et al., 1997). In plants, its mutation has been shown to affect the development of complex cell shapes such as trichomes and pavement cells (Le et al., 2003, 2006; Mathur et al., 2003a, b; Schwab et al., 2003; Saedler et al., 2004; Djakovic et al., 2006). Whereas the role of Arp2/3 complex in trichome shape development is the polarization of actin filaments for proper cell wall building (Yanagisawa et al., 2015), the role of Arp2/3 complex in pavement cell shape determination is not fully understood. Previous analysis has shown expression of ARP2 in all plant tissues with predominant expression in vascular tissues (Klahre and Chua, 1999). However, little is known about the expression of the other subunits and which factors affect it.

Correct cell shape formation requires the coordinated functioning of the cytoskeleton and hormone signaling. Auxin has been shown to be a major hormone involved in cell shape and patterning (Teale et al., 2006; Gallavotti, 2013; Saini et al., 2013). Auxin-driven cell morphogenesis relies in the correct localization of auxin carriers, which regulate this hormone’s cell-to-cell transport in order to create different concentration gradients, and failure to efficiently transport auxin across cells results in reduced tissue differentiation (Lacek et al., 2017). Therefore, correct auxin transporter localization is of utmost importance for correct auxin distribution.

It has been reported that the actin cytoskeleton has a role in auxin carrier distribution, and its disturbance results in delocalized PIN transporters (Yamamoto and Kiss, 2002; Hou et al., 2003; Lanza et al., 2012). Previous work has revealed that the lack of functional Arp2/3 complex affects auxin transporter localization leading to reduced basipetal and radial transport in mature stems. Also, issues in the generation of proper auxin maxima have been observed in these mutant lines (Sahi et al., 2018).

Our work aims to resolve in higher detail the involvement of the Arp2/3 complex in auxin signaling. For this, we analyzed Arp2/3 complex subunit expression patterns and the effect of auxin on their transcription, demonstrating that two Arp2/3 subunits’ expression is sensitive to auxin. Focusing on cotyledon pavement cells as a morphogenetic model, we analyzed the role of Arp2/3 complex in auxin-driven cell expansion through PIN3-mediated auxin transport. Our results indicate increased PIN3 targeting to vacuoles in early phases of pavement cells expansion and changed auxin balance in plants lacking functional Arp2/3 complex. Since we also demonstrate that Arp2/3 mutant pavement cells show defect in vacuolar fusion, we hypothesize that the loss of Arp2/3 results in endomembrane trafficking regulation defects, which lead to defects in precise timing of auxin transporters targeting. Our results indicate that correct morphogenesis relies in the coordinated action of auxin and the Arp2/3 complex.

## Materials and Methods

### Plant Material and Growth

Plants were grown in peat pellets or *in vitro* (vertical agar plates containing half-strength Murashige and Skoog medium supplemented with 1% w/v sucrose) under a photoperiod of 16h light:8h darkness and 23°C and light intensity 110 μmol/m^2^/s.

*Arabidopsis thaliana* genotypes used in this study were Col-0 (wild-type), *arp2* (SALK_077920.56.00), *arpc4* (SALK_013909.27.65), *arpc5* (SALK_123936.41.55), and *yuc1D* (Zhao et al., 2001).

The reporter line *pPIN3::PIN3:YFP* (Žádníková et al., 2010), *DII-VENUS* (Brunoud et al., 2012) and *yuc1D* were crossed to *arpc5*, and *γTIP-mCherry* (ABRC stock #CD3-975; Nelson et al., 2007) was crossed to *arp2*, *arpc4* and *arpc5*. Then, the F_2_ generation was screened for homozygous *arpc5* mutants expressing the reporter or showing the *yuc1D* phenotype. For *pPIN3::PIN3:YFP* and *arpc5* crosses, three independent homozygous lines (L1-L3) were used in this study.

### Auxin Treatment

Three-day-old seedlings were transferred to 1 ml of liquid half-strength Murashige and Skoog medium supplemented with 1% w/v sucrose. Plants were supplied with either IAA (5 μM, Sigma #I2886), NAA (5 μM, Sigma #N0640) or DMSO (0.1%) and cultivated for 48 h in the cultivation room with mild shaking.

For histochemical promoter-GUS activity, three-day-old seedlings were submerged in 1 μM NAA-containing liquid medium for 24 h.

### Auxin Metabolic Profiling

Auxin and its conjugates were measured in 14 DAG seedlings of Col-0 and *arp2, arpc4* and *arpc5* lines. Approximately 100 mg of fresh plant material were frozen in liquid nitrogen and stored at −80°C until analysis. Samples were analyzed as described in Dobrev and Vankova (2012). Three biological replicates were performed.

### Cloning and Plant Transformation

To generate the promoter::GUS reporter lines we amplified arbitrarily 1–2 kbp promoter regions from Col-0 genomic DNA as described in Table 1.

**TABLE 1:** List of primers used for promoter activity analysis.

Vector name	Positions (relative to start codon)	Product size	Primer sequence
pARP2	−3 to −1347 bp	1344 bp	PARP2-F 5′-AAGCTTTAACTGT GGGAAGGTTTTGAACTAG-3′
			PARP2-R 5′-GGATCCTCTC CGATTTCTATAGAGACTACAGA-3′
pARPC3	−16 to −1173 bp	1157 bp	PARPC3-F 5′-AAGCTTTGTTTTT ACGACATGAAGGGTTTC -3′
			PARPC3-R 5′-GGATCCACAAT GAAGCGATATCAGGAAGGA-3′
pARPC4	2 to −1655 bp	1657 bp	PARPC4-F 5′- AAGCTTTTCGTC CTGTTCCATCATCAAAG-3′
			PARPC4-R 5′-GGATCCATGTCCTAGAAA TGATGTTATTCTACTC-3′
pARPC5	−3 to −1420 bp	1417 bp	PARPC5-F 5′- AAGCTTCAACCACATCT CCAACTTTTCAG-3′
			pARPC5-R 5′- CTGCAGTCGATTCGATC TTTCTCTCCGA-3′

The resulting fragments were cloned into pDrive (#231124, Qiagen, Hilden, Germany) and sequenced. Subsequently, the fragments were transferred to the binary vector pRD410 (Datia et al., 1992).

Four to five-week-old Col-0 plants were transformed according to the modified floral dip method described in Narusaka et al. (2010). T2 progeny of independent transformants were tested for GUS staining and representative lines with stronger GUS intensity were used in further experiments.

### Histochemistry

Whole seedlings were harvested and incubated immediately in ice-cold 90% acetone for approximately 30 min. Then, plants were washed twice in phosphate buffer (280 mM KH_2_PO_4_, 720 mM K_2_HPO_4_, pH 7.2). Subsequently, they were incubated in GUS staining buffer (0.1 M phosphate buffer, 0.5 mM K_3_[Fe(CN)_6_], 0.5 mM K_4_[Fe(CN)_6_], 2 mM X-Gluc 5-Bromo-4-chloro-3-indoxyl-beta-D-glucoronide) at 37°C until the blue stain was visible (30 min to overnight). Seedlings were transferred to 70% EtOH and subsequently observed using an Olympus Provis AX 70 transmitted light microscope.

### Immunostaining

Longitudinal sections of 5-week-old *A. thaliana* stems were hand-sectioned with a help of a razor blade. The obtained material was submerged in EM grade 4% paraformaldehyde in aqueous solution (PFA, Electron Microscopy Sciences #15714) in MTSB (50 mM PIPES, 5 mM EGTA, 5 mM MgSO_4_; pH = 6.8) and fixed in a vacuum desiccator for one hour (pressure: 500 hPa). Samples were washed 5 times in MTSBT (0.1% Triton X-100 in MTSB) for 15 min. After this, samples were washed 5 times in 0.1% triton X-100 in water for 15 min and subsequently incubated in a solution of 0.05% pectolyase in 0.4 M mannitol in MTSBT at 37°C for 30 min. Samples were washed 5 times in MTSBT for 15 min, and 2 times in 10% DMSO/3% IGEPAL CA-630 in MTSBT for 30 min. Sections were washed 5 times in MTSBT for 5 min and incubated in 2% BSA in MTSBT for 1 h. Samples were transferred to a 2% BSA solution in MTSBT containing goat polyclonal anti-PIN1 aP-20 (1:500, Santa Cruz Biotechnologies #sc-27163) and incubated at 37°C for 4 h. Stems sections were washed 8 times in MTSBT for 15 min and then incubated for 3 h at 37°C in 2% BSA in MTSBT with secondary antibody Alexa Fluor 488 mouse anti-goat (1:1000, Abcam #ab150113). Samples were washed 5 times in MTSBT and 5 times in water for 15 min and transferred to a 0.02% sodium azide in 50% glycerol until observation under confocal microscope. All steps were performed at RT if not stated otherwise. Immunostaining was done in three biological replicates.

### qRT-PCR

Total RNA was extracted from 5DAG seedlings using the NucleoSpin^®^ RNA Plant Kit (#740949, MACHEREY-NAGEL GmbH & Co., KG, Düren, Germany). 1 μg of RNA was additionally treated with DNase I (#EN0525, Thermo Fisher Scientific). After this, cDNA was synthetized using RevertAid reverse transcriptase (#K1691, Thermo Fisher Scientific) and Oligo(dT) primers. Quantitative PCR was performed using the Light Cycler 480 instrument (Roche Applied Science, Mannheim, Germany). Reaction mixture contained 5 μl iQ^TM^ SYBR Green Supermix (#1708882, BioRad, Irvine, CA, United States), 0.2 μl of 0.01 mM primers (Table 2) and 1 μl of 2.5X diluted cDNA in a final volume of 10 μl. Cycling conditions were as follows: initial denaturation for 3 min at 94°C was followed by 50 cycles of 15 s at 94°C, 10 s at 58°C, and 20 s at 72°C. Samples were measured in triplicates for three biological replicates and RNA samples as well as the premixes alone were used as negative control. Amplification efficiencies were estimated using LinRegPCR software (Ramakers et al., 2003). The relative expression of a target gene was calculated using **Equation 1**.

**TABLE 2:** List of primers used for qRT-PCR analysis of subunit expression.

Primer name	Sequence (5′→ 3′)
ARP2-F	ACCATGTACCCAGGATTACC
ARP2-R	CGATCCGCAATCTGAGTTTC
ARP3-F	AAATTACGTCTCAACCGGTGGA
ARP3-R	CAACTCGCGAAAAACTCAGGAG
ARPC1A-F	TCTCTGTCCTAACAACACTGA
ARPC1A-R	CGATTTTGTTGGACTTTGAGC
ARPC2A-F	TAGAGAAGTGGTGATGGGTG
ARPC2A-R	AGTGACTTTATCCGCCTGAG
ARPC3-F	CTCCTTCCTGATATCGCTTC
ARPC3-R	AAGGTGATTGCCTCGTCTAC
ARPC4-F	TAAGTCTGGTGCAAGTCTCG
ARPC4-R	TTCTGTAAGCACATGGCAGC
ARPC5-F	AATCGAGGAAGATTGAAAGCC
ARPC5-R	CGACATCAAGAGCATTGAGC
EF1α-F	TGAGCACGCTCTTCTTGCTTTCA
EF1α-R	GGTGGTGGCATCCATCTTGTTACA
UBC9-F	GCTCTCACAATTTCCAAGGTGCTGC
UBC9-R	AGGGTCCTTCCTTAAGGACAGTATTTGTG

(1) $$\frac{E_{r\,e\,f}^{C_{P}^{r\,e\,f}}}{E_{t\,a\,r\,g\,e\,t}^{C_{P}^{t\,a\,r\,g\,e\,t}}}$$

where E_ref_ and E_target_ correspond to the PCR efficiencies of the reference and target genes, respectively, and $C_{P}^{\text{ref}}$$C_{P}^{\text{target}}$ correspond to the crossing points.

### Pavement Cell Analysis

Cotyledons of seedlings grown for 5 days (treated and untreated) were incubated for 20-30 min in an aqueous solution of propidium iodide (PI) of a final concentration of 0.01 mg/ml. Pavement cell shape parameters were measured using Fiji platform (Schindelin et al., 2012). Pavement cell shape analysis was carried out as described in Sahi et al. (2018). The analysis was performed in three biological replicates.

### DII-VENUS Quantification

Col-0/*DII-VENUS* and *arpc5*/*DII-VENUS* were measured at 1DAG. Seedlings were incubated in a 0.01 mg/ml PI solution for 10 min and taken for observation under confocal microscope. Images were processed using the Fiji platform (Schindelin et al., 2012). Nuclear DII-VENUS signal was quantified in the slice with higher fluorescence intensity in individual pavement cells, corrected for background signal and normalized to guard cell fluorescence intensity. Values are represented relative to the wild-type control. Samples were measured in three independent biological replicates.

### FRAP Analysis

For FRAP experiments, we employed the Zeiss LSM880 confocal microscope with C-Apochromat 40×/1.2 W Corr FCS M27 objective. The experiment was performed in two independent lines in three biological replicates. For bleaching, a region of interest was chosen at the transversal plasma membrane of pavement cells. Bleaching with 80% laser intensity was followed by tracking fluorescence recovery for approximately 159 s capturing an image every 1.1 s. To compensate for the fluorescence bleaching during recovery image acquisition, an additional non-bleached ROI was applied and values on the bleached ROI were corrected for this background. Data analysis, curve fitting and parameter estimation were done using the SigmaPlot software (Systat Software, San Jose, CA, United States). PIN3-YFP was assumed to freely diffuse in the plasma membrane, therefore a simple exponential equation (**Equation 2**) was used to fit the normalized FRAP curve.

(2) $$I\,\left(t\right)=A\,\left(1-e^{\tau \,t}\right)$$

where A corresponds to the mobile fraction or end value of the recovered intensity, t is time and τ is the fitted parameter. The latter one was next used to determine the halftime of the recovery by the following equation (**Equation 3**).

(3) T12=In0.5−τ

### PIN3-YFP Localization and FM4-64 Co-localization

*pPIN3::PIN3:YFP* seedlings were used for measurement of PIN3-YFP intensity at the plasma membrane in 1-day-old seedlings. After stratification for 2 days at 4°C, plates were transferred to the cultivation room for 24–48 h. Only those seedlings that had emerged from the seed coat in the stage before cotyledon greening were used for observation and subsequent quantification. The intensity was measured as the mean gray value for the areas representing the plasma membrane and within the cotyledon pavement cells. For colocalization of FM4-64 and PIN3-YFP, Col-0/*pPIN3::PIN3:YFP* and *arpc5/pPIN3::PIN3:YFP* seedlings were grown in 0.5 ml of liquid half-strength Murashige and Skoog medium supplemented with 1% w/v sucrose on a horizontal shaker (slow agitation at 50 rpm) for 24 h. Subsequently, FM4-64 was added to a final concentration of 4 μM in a water solution and seedlings were cultivated overnight on a horizontal shaker (50 rpm). Cotyledons were observed using the Zeiss LSM880. Images were processed using the Fiji platform (Schindelin et al., 2012).

### Vacuole Shape Analysis

Seedlings harboring the *γTIP-mCherry* reporter were grown for 6 days and adaxial cotyledon pavement cells were observed immediately under the Leica TCS SP8 confocal microscope. Alternatively, seedlings grown for 5, 9, or 14 days were incubated overnight in 4 μM FM4-64 water solution and adaxial cotyledon surface was observed under the Leica TCS SP2 confocal microscope.

Image analysis was carried out using the Fiji platform (Schindelin et al., 2012). Mid-plane of pavement cell was used for measurements. A square region of approximately 600 μm^2^ was placed framing three-way junctions of three cells. The selected areas were thresholded and binarized, delimiting the plasma membrane and tonoplast. The area occupied by the central vacuole was measured in relation of the total size of the selected region and represented as a percentage (vacuolar occupancy). Three biological replicates were performed.

### Confocal Microscopy

Zeiss LSM880 with C-Apochromat 63×/1.2 W Corr FCS M27 objective was used for the observation of *pPIN3::PIN3:YFP* (ex: 488 nm, em: 499–552 nm), FM4-64 (ex: 488 nm, em: 579–686 nm), *DII-VENUS* (ex: 488 nm, em: 499–552 nm), and propidium iodide (488 nm; em: 593–668 nm). For pavement cell shape analysis and vacuole shape analysis, propidium iodide-stained (ex: 488 nm; em: 593–668 nm) and FM4-64 (ex: 514 nm; em: 617–802 nm) stained cotyledons were observed under the laser scanning microscope Leica TCS SP2 using HC PL APO 20.0x/0.70 IMM/CORR UV objective or HCX PL APO 63.0x/1.20 W CORR UV objective, respectively. For vacuole shape analysis in seedlings harboring the *γTIP-mCherry* reporter, Leica TCS SP8 confocal microscope and HC PL APO CS2 63x/1.20 W objective, ex: 633 nm; em: 794–799 nm, was used.

## Results

### Arp2/3 Complex Subunits Are Expressed in Developing Tissues, Epidermal Cells and Vascular Tissues

**FIGURE 1:**
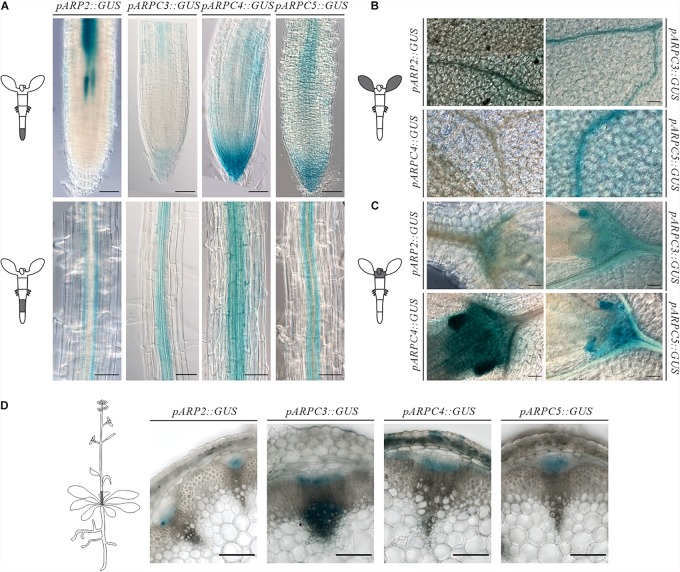
Spatiotemporal expression pattern of individual ARP2/3 complex subunits genes (ARP2, ARPC3, ARPC4 and ARPC5) as revealed by promoter-GUS activity. Analysis of GUS histochemical staining was done in *Arabidopsis thaliana* 7-day-old seedlings roots **(A)**, cotyledons **(B)**, shoot apical meristem **(C)**. Reporter signal was present at the provasculature, columella and quiescent center, pavement cells, mesophyll, stomata and shoot apical meristem **(D)** GUS production was tested in transverse sections at the base of six-week-old plants showing promoter activity in vascular bundles. Scale bar = 100 μm.

In order to pinpoint specific tissues where the Arp2/3 complex has a relevant role, we analyzed the activity of the promoter of several of its subunits fused to GUS at several developmental stages. GUS histochemical analysis of independent lines for each of the construct revealed comparable expression patterns. Generally, independent subunits showed strong expression around the vasculature tissues in both above ground tissues and roots (Figure 1). Although the signal strength was somewhat lower in pARP2::GUS line, the pattern was found to be similar for all tested lines.

Concerning the root, the reporter presence was always detected in root columella and lateral root cap, more prominently for the Arp2/3 complex subunits *ARPC3*, *ARPC4*, and *ARPC5.* Expression was also observed around the vascular tissues in the root elongation zone for all studied subunits (Figure 1A). Root cross-section of GUS-stained pARP2::GUS line allowed us to localize the GUS signal to phloem cells of the vascular bundle (Supplementary Figure 1). Further, the reporter was detected in root epidermal cells in a discontinuous pattern. The promoter activity in phloem in the vasculature was first detected in the root elongation zone (Figure 1A) and was detectable in all other parts of seedlings including the hypocotyl and cotyledons (Figures 1A,B).

In young cotyledons, all analyzed Arp2/3 subunits were expressed on varying levels (Figure 1B). Also here, stronger signal was always observed around the vasculature. However, other cell types such as mesophyll, pavement cells and stomata showed promoter activity as well. This is consistent with previously described phenotypes of Arp2/3 mutants, which include changed morphology of pavement cells.

All studied subunits showed strong expression in the shoot apical meristem and developing leaves. Higher levels of expression were observed in stipules, which have been associated with leaf vascular development (Aloni et al., 2003; Cheng et al., 2007) (Figure 1C).

In 6-week-old inflorescences, β-glucoronidase activity was detected in procambium and protophloem tissues as well as in metaxylem for all studied subunits (Figure 1D). Interestingly, expression levels were not detected equally in all vascular bundles within the same plant. This may suggest the need of higher Arp2/3 complex activity in certain developmental situations, such as developing/differentiating vascular tissues, and its decline in already differentiated tissues.

These results indicate that Arp2/3 subunits are required especially in developing tissues (root and shoot apical meristem) and in epidermal cells. The GUS reporter further showed characteristic expression in vascular bundles.

### Arp2/3 Subunits’ Expression Is Stimulated by Auxin

**FIGURE 2:**
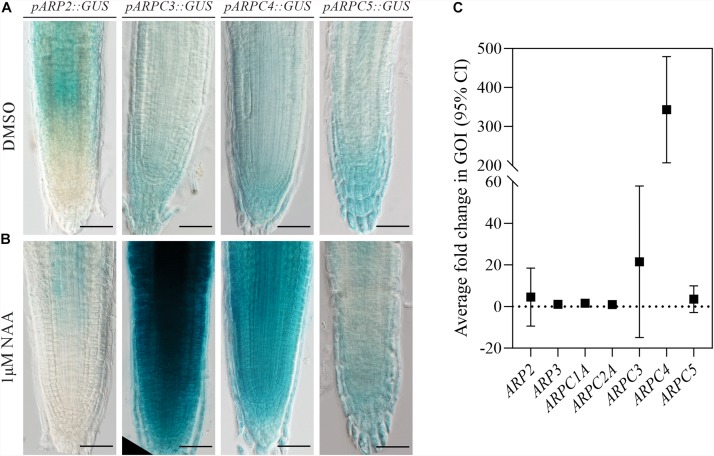
ARP2/3 subunit expression is stimulated by increased auxin levels. Plants expressing the GUS reporter gene under the control of individual ARP2/3 complex subunits’ promoters (pARP2, pARPC3, pARPC4 and pARPC5) were stained for GUS activity after 24-h treatment with 0.1 % DMSO **(A)** or 1 μM NAA **(B)**. **(C)** Relative transcription levels of individual complex subunits were quantified in five-fay-old *YUC1D* seedlings by qRT-PCR in three biological replicates. Scale bar = 100 μm).

Previously, the Arp2/3 complex has been shown to have a role in auxin distribution (Sahi et al., 2018). To investigate the importance of auxin in regulating individual subunit expression, we characterized their expression after the increase of auxin levels. For this, generated transgenic lines carrying promoter fusions were subjected to NAA treatment. Strikingly, just two of the subunits analyzed (*AtARPC3* and *AtARPC4*) showed increased promoter activity when compared to the control treatment (Figures 2A,B).

To test the susceptibility of expression of Arp2/3 subunits to auxin under conditions more physiological than external auxin application, we used the previously described dominant gain-of-function auxin over-production mutant *YUC1D* (Zhao et al., 2001), which shows increased free IAA endogenous level when compared to wild-type plants, to analyze the expression of Arp2/3 subunits. qRT-PCR analysis confirmed the previous result, because only *AtARPC3* and *AtARPC4* exhibited higher RNA levels in *YUC1D* line when compared to wild type (Col-0) (Figure 2C).

Taken together, two different methods demonstrated that the transcription of *ARPC3* and *ARPC4* genes coding Arp2/3 complex subunits was enhanced in the presence of higher auxin levels, while other subunits showed no significant change.

### Auxin Aggravates Pavement Cell Morphology Defects in Plants Lacking Functional Arp2/3 Complex

Along with distorted shape of leaf trichomes, pavement cell shape change is one of the first described phenotypes of plants with dysfunctional Arp2/3 complex (Deeks and Hussey, 2003). Since all mutant lines lacking either one of the Arp2/3 subunit or Arp2/3-activation complex subunits show this characteristic phenotype, it is considered as a typical phenotype of Arp2/3 mutants (Li et al., 2003; Mathur et al., 2003a, b; Djakovic et al., 2006). Auxin has also been long discussed to play a role in pavement cell interdigitation (Li et al., 2011; Xu et al., 2014; Gao et al., 2015; Belteton et al., 2018). We therefore decided to use pavement cell shape as a morphogenesis model to answer the question of whether auxin and Arp2/3 were connected in the cell shape control.

**FIGURE 3:**
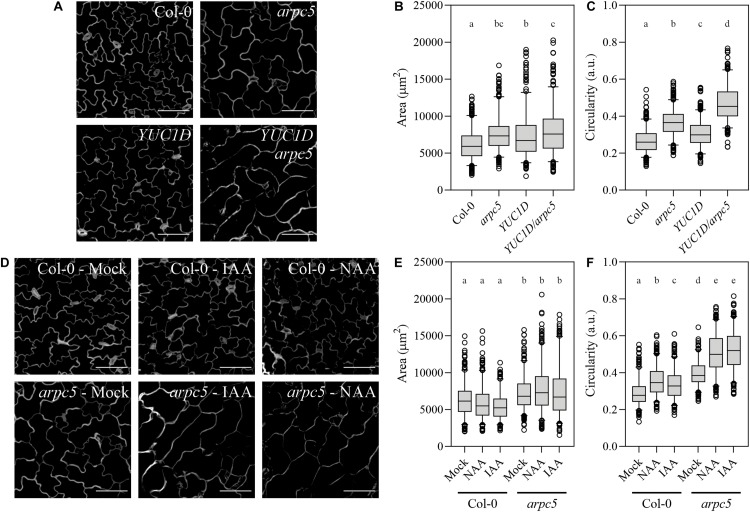
Pavement cells morphology upon increased auxin levels. **(A–C)** The effect of increased endogenous auxin level in *YUC1D* plants crossed with *arpc5.* Representative pictures **(A)** and quantitative cell shape analysis **(B,C)** of five-day-old Col-0, *arpc5*, and *YUC1D, YUC1D/arpc5* epidermal lobed cells is shown. **(D–F)** The shape phenotype observed in *YUC1D* and *YUC1D/arpc5* could be mimicked by 48 h treatment with 5 μM auxin (IAA and NAA) of three-day-old Col-0 and *arpc5* seedlings. Illustrative pictures are shown in **(D)**, quantitative cell shape analysis is depicted in **(E)** and **(F)**. Scale bar = 100 μm. *n* = 250–350 cells, three biological replicates (Tukey HSD, *p* < 0.001; *p* < 0.05 for the area comparison between *YUC1D* and *YUC1D/arpc5*).

We crossed the *arpc5* knockout plants with the previously mentioned auxin over-producing line *YUC1D*. Double homozygous lines were selected, and pavement cell size and shape parameters were determined. Both single and double mutant visibly showed larger cells and reduced cell complexity (Figure 3A), represented by increased area (Figure 3B) and higher circularity values (Figure 3C), which is consistent with known phenotypes of Arp2/3 mutant plants and plants with increased auxin level. However, *YUC1D/arpc5* exhibited a more dramatic defect in lobe formation than single mutants, suggesting an interaction between the two pathways (Figures 3B,C). This observation was further confirmed in our experiments, where auxin was applied externally to pavement cells. The application of 5 μM IAA or NAA to *arpc5* mutant for 48 h mimicked the phenotype of *YUC1D/arpc5* plants, because the formation of lobes was reduced after treatment with auxins (Figure 3D). No effect of auxin treatment was observed in wild-type or *arpc5* plants. Changes in cell area were only due to the lack of functional Arp2/3 complex (Figures 3E,F).

Our results confirmed cell expansion defect in plants lacking Arp2/3 complex, and an additive effect on cell shape in Arp2/3 mutant with increased auxin level.

### Elevated Levels of oxIAA and IAA Marker in Cotyledon Pavement Cells Suggest Altered Auxin Balance in Cells of Arp2/3 Mutants

Since auxin has an effect in pavement cell morphogenesis and Arp2/3 mutants seem to show an enhanced response to increased levels of this hormone, we sought to determine whether auxin levels were affected in Arp2/3 complex mutants. We analyzed endogenous auxin levels in seedlings of wild-type and *arpc5*, *arpc4* and *arp2* mutants. The biochemical analysis of active IAA suggests that mutant plantlets contain IAA levels comparable to those found in wild type. However, a clear increase of oxIAA-GE levels in mutants (considered to be an inactive form of auxin, Pénčík et al., 2013) was repeatedly and consistently detected in all tested Arp2/3 mutant lines (Table 3).

**TABLE 3:** Quantification of endogenous auxin levels in Arabidopsis thaliana seedlings.

	IAA		oxIAA-GE	
		
	Above ground tissues	Roots	Above ground tissues	Roots
Col-0	68.35 ± 3.79	122.12 ± 23.07	886.02 ± 298.11	1102.03 ± 552.34
*arp2*	60.48 ± 3.58	162.27 ± 31.57	1517 ± 266.58	3739.72 ± 1747.12
*arpc4*	50.22 ± 7.57	143.64 ± 18.20	1307.14 ± 133.04	3953.52 ± 2239.16
*arpc5*	84.69 ± 19.13	130.55 ± 35.01	1892.99 ± 576.35	3450.99 ± 2269.34

*Values in pmol/gFW; average of three biological replicates +/− SD.*

**FIGURE 4:**
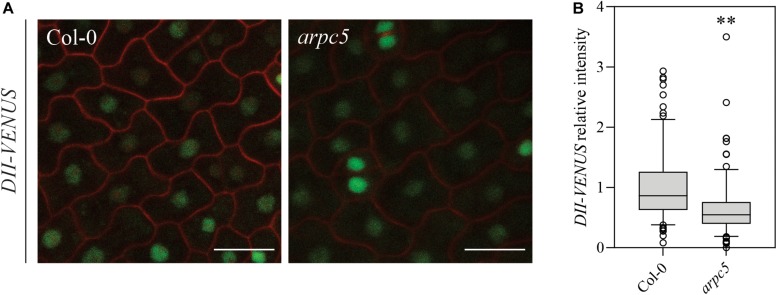
Plants lacking functional ARP2/3 complex show increased active auxin-driven proteasomal degradation of the auxin input sensor DII-VENUS. **(A)** *In vivo* observation of *DII-VENUS* (green) in one-day-old wild-type and *arpc5* seedlings (scale bar = 20 μm) co-stained with PI (red). **(B)** Normalized DII-VENUS relative intensity (*n* = 167 cells; three biological replicates; Student’s *t*-test, *p* < 0.0001).

This prompted us to analyze auxin-driven proteasomal degradation of DII-VENUS marker in individual cells of cotyledon epidermal lobed cells. We generated *arpc5* crosses with DII-VENUS and analyzed nuclei fluorescence using confocal microscopy (Figure 4A). Interestingly, *arpc5* was the only Arp2/3 mutant line successfully crossed with this marker. When compared to Col-0 carrying DII-VENUS, *arpc5* plants displayed a significant decrease of DII-VENUS signal (Figure 4B). This result indirectly suggests that there is either a mild increase in auxin levels or increased auxin response in *arpc5*.

Altogether, the present data show that Arp2/3 mutants could have mildly increased concentration of intracellular auxin in pavement cells, and that general balancing of auxin concentration in tissues results in higher levels of inactivated auxin, suggesting that the Arp2/3 complex is important to maintain the correct auxin balance at the cellular and tissue level.

### PIN3 Does Not Show Mobility Defects in the Plasma Membrane

**FIGURE 5:**
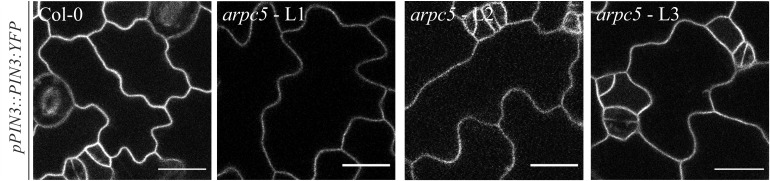
*In vivo* observation of *pPIN3::PIN3:YFP* in three-day-old *arpc5* plants. L1, L2 and L3 correspond to three independent crosses between *pPIN3::PIN3:YFP* and *arpc5* lines used in this study (scale bar = 20 μm).

**FIGURE 6:**
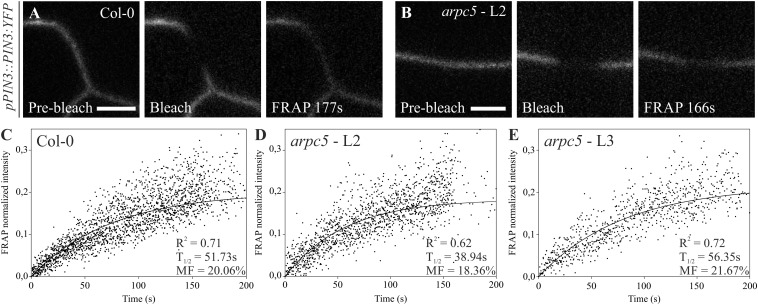
Fluorescence recovery after photobleaching (FRAP) of *pPIN3::PIN3:YFP* auxin carrier in Col-0 **(A,C)** and *arpc5* lines L2 **(B,D)** and L3 **(E)**. Photobleaching followed by its recovery was analyzed in transversal PM (plasma membrane) of three-day-old cotyledons expressing PIN3-YFP. Simple exponential fit was applied to normalized FRAP data; individual fluorescence intensities for multiple plants (*n* = 6–14) are shown (scale bar = 5 μm). The analysis was performed in three biological replicates.

PIN protein dynamics in the plasma membrane has been shown to be important for the proper regulation of auxin homeostasis (Geldner et al., 2001; Abas et al., 2006; Kleine-Vehn et al., 2011), and the cytoskeleton has been shown to be involved in these processes (Geldner et al., 2001; Friml et al., 2002; Rahman et al., 2007; Kleine-Vehn et al., 2008b; Lanza et al., 2012). PIN3 is one of the predominant PINs expressed in pavement cells (Le et al., 2014). We therefore analyzed PIN3-YFP localization in pavement cells and tested the mobility of PIN3 in the plasma membrane of pavement cells in three-day-old seedlings by FRAP (Fluorescence Recovery After Photobleaching, Figures 5, 6). Our results show that the localization of PIN3-YFP was not changed in *arpc5* mutants (Figure 5). Likewise, the mobile fraction and halftime recovery time values (Figures 6D,E) in *arpc5* plants were comparable to those observed in wild-type (Figure 6C). Although it has been reported to be Brefeldin A (BFA) sensitive (Friml et al., 2002), the analysis of PIN3 cycling between endosomes and plasma membrane in cotyledon pavement cells using this inhibitor treatment was not possible, because PIN3 localization in pavement cells was not sensitive to BFA and no BFA-compartment was observed (data not shown). This indicates that PIN3 localization and lateral membrane dynamics are not changed in 3-d-old cotyledons of plants lacking functional Arp2/3 complex.

### Precise Localization of PIN3 and PIN1 Is Inefficient in Mutants Lacking Functional Arp2/3 Complex

**FIGURE 7:**
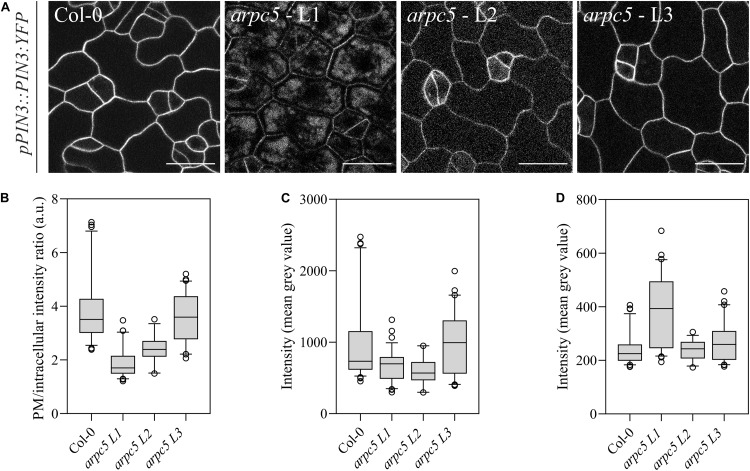
Plants lacking functional ARP2/3 complex show altered PIN3 localization. **(A)** *In vivo* observation of *pPIN3::PIN3:YFP* in one-day-old plants was altered in three independent crosses between *pPIN3::PIN3:YFP* and *arpc5* lines (L1-3); scale bar = 20 μm. **(B)** The ratio between PM and intracellular PIN3-YFP intensity is reduced *arpc5* mutants. **(C,D)** PIN3-YFP intensity was measured as the mean gray value for the areas representing the plasma membrane **(C)** and within the cell **(D)**. Results show that the differences in ratio are due to increased intracellular signal. *n* = 45–70 cells, three biological replicates (Tukey, HSD *p* < 0.001).

It has been previously reported that pavement cell shape determination occurs predominantly within the first two days after germination (Zhang et al., 2011; Armour et al., 2015; Wu et al., 2016). We investigated whether Arp2/3 takes part in PIN3 localization at these initial steps of epidermal cell morphogenesis. Indeed, when observing one-day-old plants, a decrease in the *pPIN3::PIN3:YFP* intensity ratio between the plasma membrane and the cell interior was detected (Figures 7A,B). Absolute intensity values indicate that the reason for this decrease in the ratio is a result of reduced amounts of PIN3 at the plasma membrane and increased intracellular signal (Figures 7C,D).

In our previous work we demonstrated that auxin transport in stems is very limited in plants lacking functional Arp2/3 complex (Sahi et al., 2018). As PIN1 is the main auxin transporter mediating basipetal polar auxin transport through plant tissues (Gälweiler et al., 1998), we assayed if also PIN1 localization is affected in mutants plants’ stems. Consistent with our previous observations, analysis of longitudinal stem sections with immunolocalized PIN1 showed localization to basal membranes of elongated parenchyma cells in wild-type plants. Basal localization of PIN1 was disturbed in Arp2/3 mutant lines, where signal was found also on adjacent lateral membranes, demonstrating inefficient polar localization of PIN1 here (Supplementary Figure 2).

Taken together, our results demonstrate the contribution of Arp2/3 complex in PIN3 localization to the plasma membrane in very early stages of pavement cells development. Our finding of PIN1 inefficient polar localization also point on a broader need of the Arp2/3 complex in maintaining efficient PIN localization throughout plant growth.

### Plants Lacking a Functional Arp2/3 Complex Show Altered Vacuolar System

**FIGURE 8:**
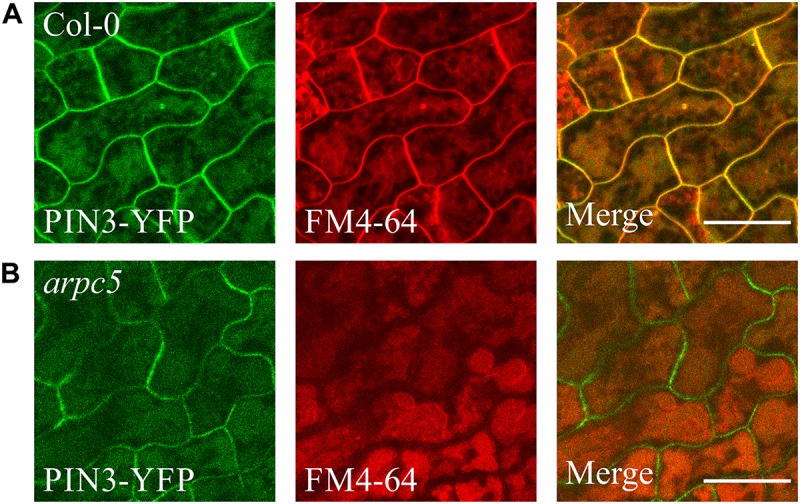
PIN3-YFP localization to the vacuole in 3DAG wild type **(A)** and *arpc5* **(B)** seedlings. 2DAG plants expressing *pPIN3::PIN3:YFP* were incubated in darkness overnight in a solution of 4 μM FM4-64 to allow for vacuolar staining (scale bar = 20 μm).

**FIGURE 9:**
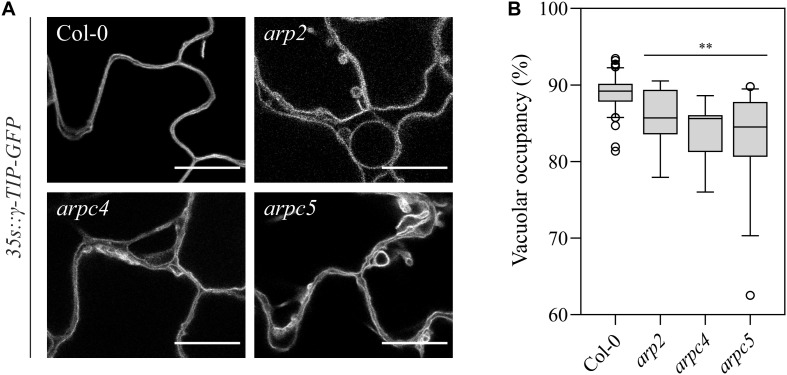
**(A)** Observation of vacuolar organization in wild-type and *arp2, arpc4* and *arpc5* in 6DAG cotyledon expressing the *γTIP-mCherry* marker (scale bar = 20 μm). **(B)** Vacuolar occupancy quantifications at three-way cellular junctions. (Analysis performed in three biological replicates; Student’s *t*-test; ^∗∗^*p* < 0.01; ^∗^*p* < 0.05).

The staining with FM4-64 confirmed that the compartments with accumulated PIN3-YFP are vacuoles (Figure 8). FM4-64 limited staining of plasma membrane of *arpc5* pavement cells, observed in our experiments (Figure 8B), may indicate changed plasma membrane properties in *arpc5* line, which became visible under conditions of overnight exposure to water solution with the dye. PIN transporters are known to cycle between the plasma membrane and endosome, and they are eventually degraded in vacuoles (Kleine-Vehn et al., 2008a). Since PIN3 accumulated in vacuolar compartments, we hypothesized that general intracellular trafficking of proteins such as vacuolar targeting may be changed in *arpc5* mutant. We therefore decided to analyze vacuolar compartment in pavement cells of plants lacking functional Arp2/3 complex. A thorough analysis of vacuole occupancy revealed that vacuolar structure is affected in Arp2/3 complex mutants (Figure 9), suggesting a more fragmented architecture. Interestingly, the defect in the fusion of vacuolar membrane, manifested as fragmented vacuoles, was detectable also in later stages of cotyledon development (Supplementary Figure 3). The phenotype was hardly distinguishable in 1-day-old seedlings, where vacuoles have very complex shape in both WT and mutants due to early steps of central lytic vacuole formation (Supplementary Figure 4). The defect in central vacuole formation in pavement cells of mutants may affect the cycling and vacuolar targeting of proteins such as early development-associated PIN3 targeting to the vacuole and plasma membrane.

## Discussion

Arp2/3 complex has been shown to be involved in numerous stages of plant development such as pavement cells, stomata and trichomes morphology, or stem thickness and cell wall quality (Le et al., 2003; Li et al., 2003; Mathur et al., 2003a, b; Brembu et al., 2004; Deeks et al., 2004; El-Assal et al., 2004; Djakovic et al., 2006; Dyachok et al., 2008, 2011; Jiang et al., 2012; Sahi et al., 2018). Promoter fusion studies have proven to be useful in hinting the importance of a protein’s function in a determined tissue or developmental stage. In our study, we aimed to determine the patterns of expression of Arp2/3 complex subunits in order to reveal the sites where their promoters are most active. Since Arp2/3 functions as a complex, we expected similar expression patterns for all tested subunits. Indeed, we can conclude that the subunits studied are expressed roughly in the same tissues during the growth of *A. thaliana*, although some minor differences can be seen in the root tip. The expression of Arp2/3 subunits was detected in most tissues reported previously to be affected in mutants lacking the active complex, including pavement cells. All subunits are shown to have prominent promoter activity in the provascular region, as it was previously described by Klahre and Chua (Klahre and Chua, 1999). Interestingly, the root cross section allowed us to localize the expression to phloem cells, while xylem precursor cell files were stained in the work of Klahre and Chua (1999). Although no severe phenotypes have been reported in vasculature in young seedlings of Arp2/3 complex mutant lines, we have previously showed that AUX1 expression in procambial and protoxylem cells in stem vascular bundles is reduced in *arpc5* line (Sahi et al., 2018). This suggests that Arp2/3 complex may be needed for vasculature development. Indeed, the cytoskeleton plays a role in its formation, as many of its mutants show vasculature formation defects (Hepler and Fosket, 1971; Falconer and Seagull, 1985; Gardiner et al., 2003; Mao et al., 2006; Oda et al., 2010; Pesquet et al., 2010; Bao et al., 2012; Oda and Fukuda, 2012, 2013; Sasaki et al., 2017; Vukašinović et al., 2017; Sugiyama et al., 2019). As the Arp2/3 complex has been reported to have a role in plant cell wall deposition (Sahi et al., 2018) and autophagy (Wang et al., 2016), we could speculate that the complex participates in the building of specialized cell walls and autophagy needed for differentiation of vascular tissue. More detailed study is needed to confirm or disprove this hypothesis.

Our previous work has shown a series of auxin-related phenotypes which include reduced auxin transporter abundance and auxin transport in mature tissues as well as reduced auxin maxima in early stage cotyledons (Sahi et al., 2018). Also, and in agreement with the expression observed through the analysis of promoter fusions, *DR5:GUS* maxima were observed to be reduced around the vascular tissue (Sahi et al., 2018). Therefore, we aimed to explore closer the role of Arp2/3 complex in auxin signaling. The presented data demonstrate that the expression of Arp2/3 complex subunits can be induced by auxin. We do not know the rationale of the selective increased transcription of only two of the subunits (*ARPC3* and *ARPC4*). Although high concentration of externally applied auxin was used in this study (1 μM), the same pattern of expression (*ARPC3* and *ARPC4*) was detected in *YUC1D* line, which contains increased endogenous level of auxin (Zhao et al., 2001). This suggests that increased expression of these two subunits reflected the response to auxin, not the stress response to externally applied high auxin concentration. The regulation of the Arp2/3 complex may involve also post-transcriptional and post-translational control mechanisms, such as mRNA or protein stability, which were not assayed here. We could hypothesize these two subunits could be important for the regulation (or trigger) of the complex assembly.

Plant Arp2/3 complex has been reported to play a role in cell expansion, possibly contributing to cell wall properties and polar growth. Especially pavement cell shape morphogenesis is studied in the context of Arp2/3 complex role, although its function is yet not well understood (Mathur et al., 1999, 2003a,b; Le et al., 2003; Li et al., 2003; Schwab et al., 2003; Deeks et al., 2004; El-Assal et al., 2004; Djakovic et al., 2006; Dyachok et al., 2008; Zhang et al., 2013; Yanagisawa et al., 2015; Sahi et al., 2018). Auxin has long been discussed to play a role in epidermal cell morphogenesis too, and has been proposed to play a role in cell wall permissivity (Xu et al., 2010, 2014; Nagawa et al., 2012; Gao et al., 2015; Belteton et al., 2018). We aimed to test the interplay between Arp2/3 and auxin in pavement cell growth. We distinguish between two processes controlling pavement cell morphogenesis: cell shape formation (cell circularity) and cell expansion (cell size). Our results revealed that an increase in auxin concentration, both constitutive (*YUC1D* line) or temporal (auxin treatment), leads to a decrease in cell shape complexity, which is more severe in the case of plants lacking a functional Arp2/3 complex. The additive effect of auxin in Arp2/3 mutant lines, as well as the fact that plants lacking Arp2/3 are still responsive to auxin, suggests partly independent functions of auxin and Arp2/3 in cell lobes formation. Cell expansion, on the other hand, is the main function of Arp2/3 complex. While *YUC1D/arpc5* line shows mildly deepened phenotype, the cell expansion of mutant line is not altered by externally applied auxin. It is important to note here that the treatment with 5 μM auxin may induce also secondary effects, which could explain the slight difference in cell expansion between *YUC1D/arpc5* and *arpc5* treated with auxin. Nevertheless, this concentration of auxin was needed to mimic *YUC1D* phenotype concerning cell shape. We can conclude that these results pinpoint the involvement of the complex in mediating the cell expansion in response to auxin.

However, our results suggest also a direct functional link between auxin and Arp2/3. The expression of Arp2/3 subunits is positively regulated by auxin, and mutants lacking Arp2/3 complex have reduced basipetal transport of auxin (Sahi et al., 2018). We also show here that Arp2/3 mutants have changed auxin metabolism in respect to increased pool of inactivated auxin, as well as increased auxin concentration in pavement cells. One of the most important factors that regulate auxin levels within and outside the cell is polar auxin transport (Lacek et al., 2017). We tested whether transporter localization was also an issue in pavement cell formation. Our previous findings on cell wall composition prompted us to first analyze whether PIN transporter dynamics were affected at the plasma membrane level, as this factor has been shown to affect plasma membrane motility of integral proteins (Feraru et al., 2011; Nakayama et al., 2012; Braybrook and Peaucelle, 2013; Ganguly et al., 2014). We show that PIN3 lateral mobility in the plasma membrane is not altered in *arpc5* mutants. However, we show that PIN3 localization is affected in *arpc5* plants at a very young stage of development, showing increased amounts in the vacuole and reduced signal at the plasma membrane. The reduction of PIN3 at the plasma membrane could result in changes in auxin homeostasis in mutant plants, which was indirectly suggested by DII-VENUS marker that showed increased auxin-driven proteasomal degradation in *arpc5* pavement cells. Of course, since we failed to generate mDII-VENUS cross with *arpc5* line, we cannot fully exclude the possibility that transcriptional activity in general or the activity of 35S promotor in *arpc5* epidermal cells is lower than in WT. It is also important to stress out that although *arpc5* line shows slightly increased auxin levels or response in pavement cells, it is still responsive to high auxin levels. Rather moderate and perhaps local or temporal increase in endogenous auxin in pavement cells of Arp2/3 mutants was further confirmed by the analysis of general auxin content in *arpc5* seedlings, because no significant increase was detected. Nevertheless, as a signaling molecule, even a small shift in endogenous auxin concentration in *arpc5* line may be physiologically relevant. In this respect, the observation that oxIAA-GE, inactivated form of auxin, is increased in three independent Arp2/3 mutant lines indeed suggests shifted auxin homeostasis. Interestingly, vacuolar localization of PIN3 is only observed at very early stages of pavement cell development. This phenomenon could be explained by several possible scenarios. The first possibility is that Arp2/3 activity is mainly required at early stages of epidermal cell morphogenesis and not later on. This would be consistent with the fact that pavement cell shape determination occurs predominantly within the first two days after germination (Zhang et al., 2011; Armour et al., 2015; Wu et al., 2016). The second hypothesis would be that at later stages, PIN3 is still localized in the vacuole, but remains unobservable due to YFP susceptibility to vacuolar pH (Kremers et al., 2016; Shinoda et al., 2018) and less concentrated amounts of PIN3 within the organelle as a result of its larger size. We could not assay PIN3 cycling between endosomal compartment and the plasma membrane, because PIN3 in pavement cells is not sensitive to BFA, a drug commonly used for this assay. Therefore, the hypothesis that PIN3 cycling between the plasma membrane and cell interior is controlled by Arp2/3 remains to be tested. However, the effect of Arp2/3 complex loss on the localization of PIN transporters may be rather general, as suggested by immunolocalization of PIN1 in stems and inefficient basal localization in parenchyma cells.

Vacuolar homeostasis is relevant in a variety of processes during plant development, ranging from turgor preservation during cell morphogenesis to protein trafficking (Krüger and Schumacher, 2018; Shimada et al., 2018). Our observation that PIN3 protein is inefficiently transported to the plasma membrane and that PIN3-YFP vacuolar concentration is increased in *arpc5* line in early stages of development pointed to the vacuolar function. In fact, vacuolar targeting depending on retromer complex function is a commonly known degradation pathway for PIN proteins (Koltzscher et al., 2003; Abas et al., 2006; Laxmi et al., 2008; Shirakawa et al., 2009; Bachmair et al., 2012; Baster et al., 2013; Belteton et al., 2018; Salanenka et al., 2018). Vacuolar shape is also modulated by auxin (Löfke et al., 2015) and in turn, vacuoles may play an important role in auxin homeostasis (Kramer and Ackelsberg, 2016). Actin cytoskeleton controls remodeling of vacuolar membranes (Zhang et al., 2014) and Arp2/3 complex has been shown to participate in vacuolar morphology control in stomata and trichomes (Mathur et al., 2003a; Saedler et al., 2004; Li et al., 2013).

Vacuolar fusion is a critical process during cell maturation and function (Viotti et al., 2013; Krüger and Schumacher, 2018). Our results point out the involvement of Arp2/3 complex in vacuolar fusion during pavement cell development as well, because two independent mutant lines (*arpc5* and *arpc4*) had fragmented vacuoles in pavement cells. The incorrect function of vacuolar compartment could be responsible for its reduced efficiency in protein recycling, being PIN3 and PIN1 an example of its consequences. Also, the lack of a larger vacuole could lead to reduced cell turgor which, in its turn, can be detrimental for cell adhesion and cell expansion itself. Interestingly, the vacuolar fusion deficiency is detectable throughout the cotyledon development.

In summary, we have shown here that Arp2/3 subunits are expressed throughout the plant tissue including pavement cells, trichomes, hypocotyls and root tips. The transcription of ARPC3 and ARPC4 subunits is positively regulated by auxin, and plants lacking functional Arp2/3 complex have increased auxin concentration in pavement cells. Investigating the relationship between auxin and Arp2/3 in pavement cell shape formation we found out that Arp2/3 complex has a restrictive role in cell expansion, which is partially independent of auxin-induced cell expansion. On the other hand, the additive effect of auxin in mutants in the formation of cell lobes suggests cooperation of Arp2/3 and auxin in the control of pavement cell shape formation. The direct interaction between auxin and Arp2/3 complex in this context may lay in the function of the complex regarding auxin transporters trafficking. Our results imply general intracellular trafficking defects in plants lacking Arp2/3 complex. This is supported by observed inefficient PIN1 polar localization in stems, inefficient PIN3 targeting to the plasma membrane, and vacuolar biogenesis defects. Altered performance of intracellular trafficking may lead to deficient auxin transport and therefore altered hormone homeostasis within single cells, contributing to the impaired cell wall remodeling that we observe in Arp2/3 complex mutants.

## Data Availability Statement

All datasets generated for this study are included in the article/Supplementary Material.

## Author Contributions

JG-G, KS, and JP conceived and designed the experiments. JG-G, KS, and IK generated the lines used in this study. JG-G, KS, ŠK, MS, and JL performed the experiments and analyzed the data obtained. JG-G and KS wrote the manuscript.

## Conflict of Interest

The authors declare that the research was conducted in the absence of any commercial or financial relationships that could be construed as a potential conflict of interest.

## Supplementary Material

FIGURE 1 Cross section of a root of pARP2::GUS line. GUS reporter was visualized in 7 DAG plants as described in Materials and methods. Roots were then embedded into 2.5 % agarose. Agarose block was fixed to a holder of vibratome and sectioned to obtain root cross sections of a thickness of 50 μm. Sections were observed using Olympus Provis AX 70 microscope. GUS reporter expressed within the vascular bundle is focused in regions corresponding to phloem cells (Dinneny and Yanofsky 2004). P, phloem; X, xylem. Scale bar = 20 μm.

FIGURE 2 **(A)** Immunostaining of PIN1 in longitudinal stem sections five-week-old wild-type plants and plants lacking functional ARP2/3 complex (*arp2, arpc4* and *arpc5*) showing disturbed localization of PIN1 at the basal end xylem parenchyma cells. Scale bar = 20 μm. **(B)** Detail of the squared area in **(A)** (scale bar = 5 μm, three biological replicates).

FIGURE 3 **(A)** Vacuolar organization in wild-type and *arpc5* plants at 5DAG, 9DAG and 14DAG. **(B)** Vacuolar occupancy quantifications at three-way cellular junctions of cotyledons stained overnight with 4 μM FM4-64 at multiple growth timepoints (5DAG, 9DAG and 14DAG) for Col-0 and *arpc5*. S (Pairwise comparisons with Student’s *t*-test; ^∗∗^*p*<0.01; three biological replicates). Scale bar = 50 μm.

FIGURE 4 Vacuolar organization in wild-type and *arp2, arpc4* and *arpc5* in 1DAG cotyledon expressing the lytic vacuole *γTIP-mCherry* marker. Scale bar = 10 μm.
