# Supplementary figures and images for: Arp2/3 Complex Is Required for Auxin-Driven Cell Expansion Through Regulation of Auxin Transporter Homeostasis
Source: Front Plant Sci. 2020 Apr 28;11:486. doi: 10.3389/fpls.2020.00486 (PMC7212389; doi:10.3389/fpls.2020.00486)

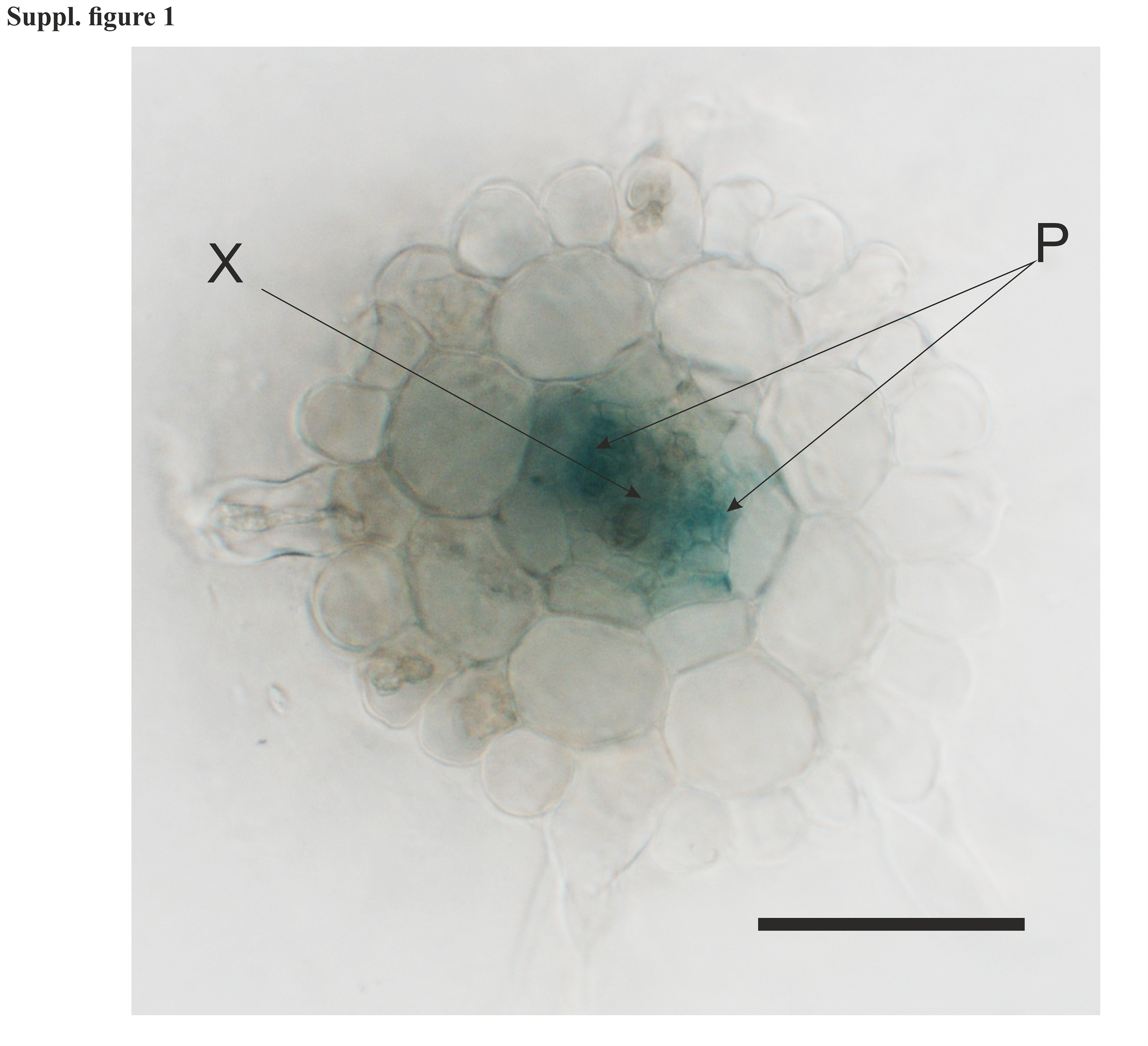

Supplement: FIGURE 1 — Cross section of a root of pARP2::GUS line. GUS reporter was visualized in 7 DAG plants as described in Materials and methods. Roots were then embedded into 2.5 % agarose. Agarose block was fixed to a holder of vibratome and sectioned to obtain root cross sections of a thickness of 50 μm. Sections were observed using Olympus Provis AX 70 microscope. GUS reporter expressed within the vascular bundle is focused in regions corresponding to phloem cells (Dinneny and Yanofsky 2004). P, phloem; X, xylem. Scale bar = 20 μm. [file Image_1.TIF]

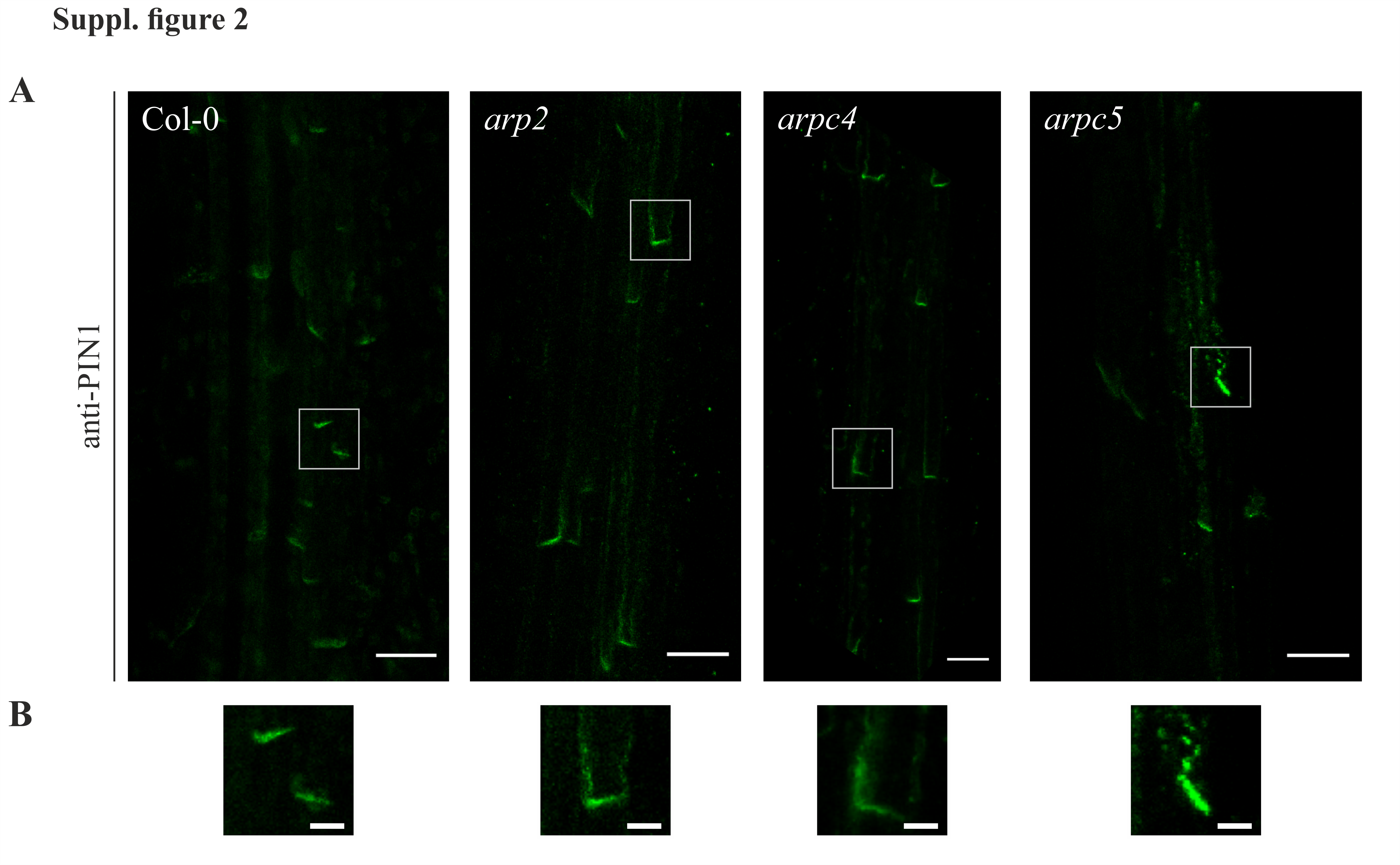

Supplement: FIGURE 2 — (A) Immunostaining of PIN1 in longitudinal stem sections five-week-old wild-type plants and plants lacking functional ARP2/3 complex (arp2, arpc4 and arpc5) showing disturbed localization of PIN1 at the basal end xylem parenchyma cells. Scale bar = 20 μm. (B) Detail of the squared area in (A) (scale bar = 5 μm, three biological replicates). [file Image_2.TIF]

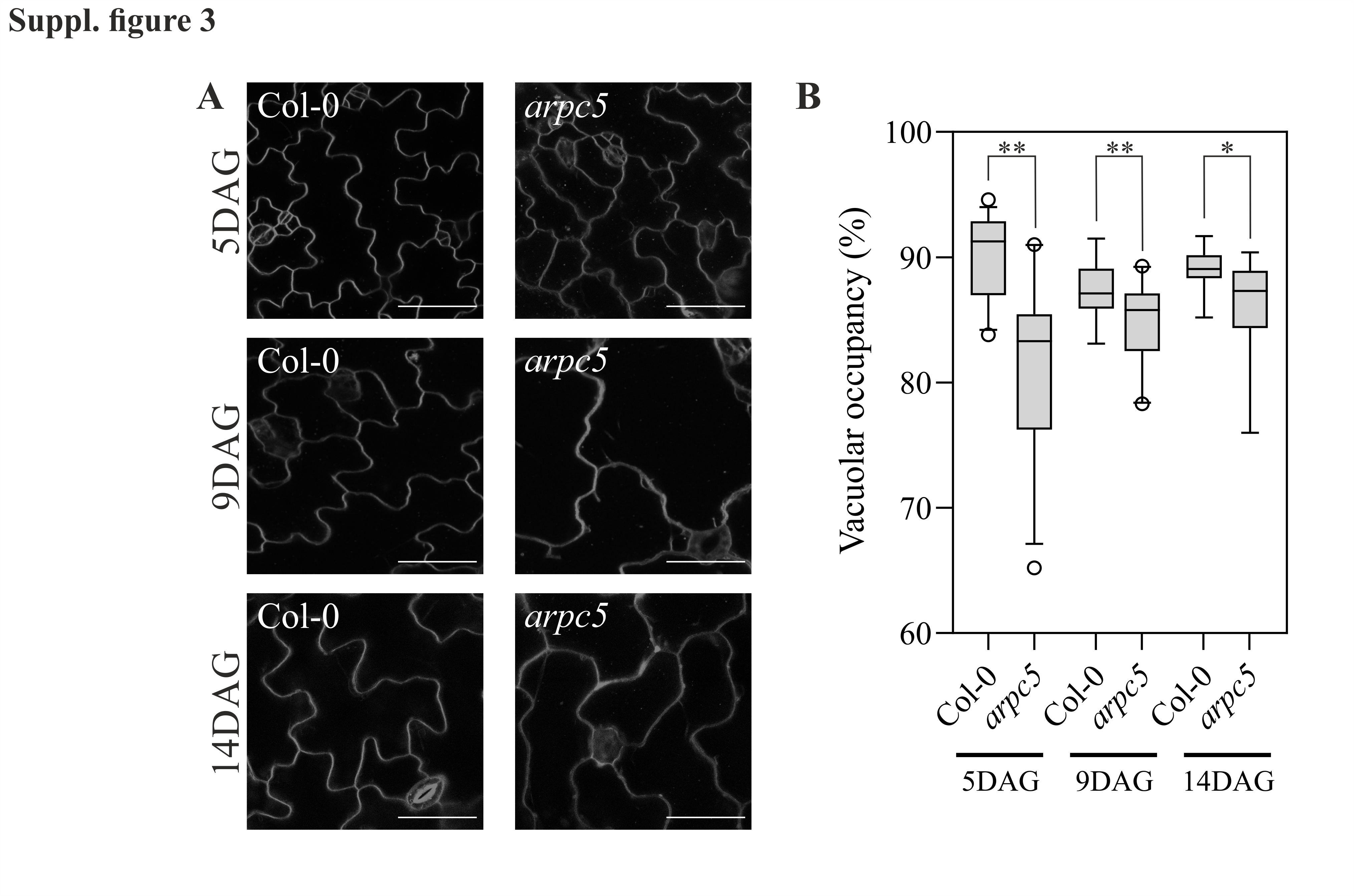

Supplement: FIGURE 3 — (A) Vacuolar organization in wild-type and arpc5 plants at 5DAG, 9DAG and 14DAG. (B) Vacuolar occupancy quantifications at three-way cellular junctions of cotyledons stained overnight with 4 μM FM4-64 at multiple growth timepoints (5DAG, 9DAG and 14DAG) for Col-0 and arpc5. S (Pairwise comparisons with Student’s t-test; ∗∗p<0.01; three biological replicates). Scale bar = 50 μm. [file Image_3.TIF]

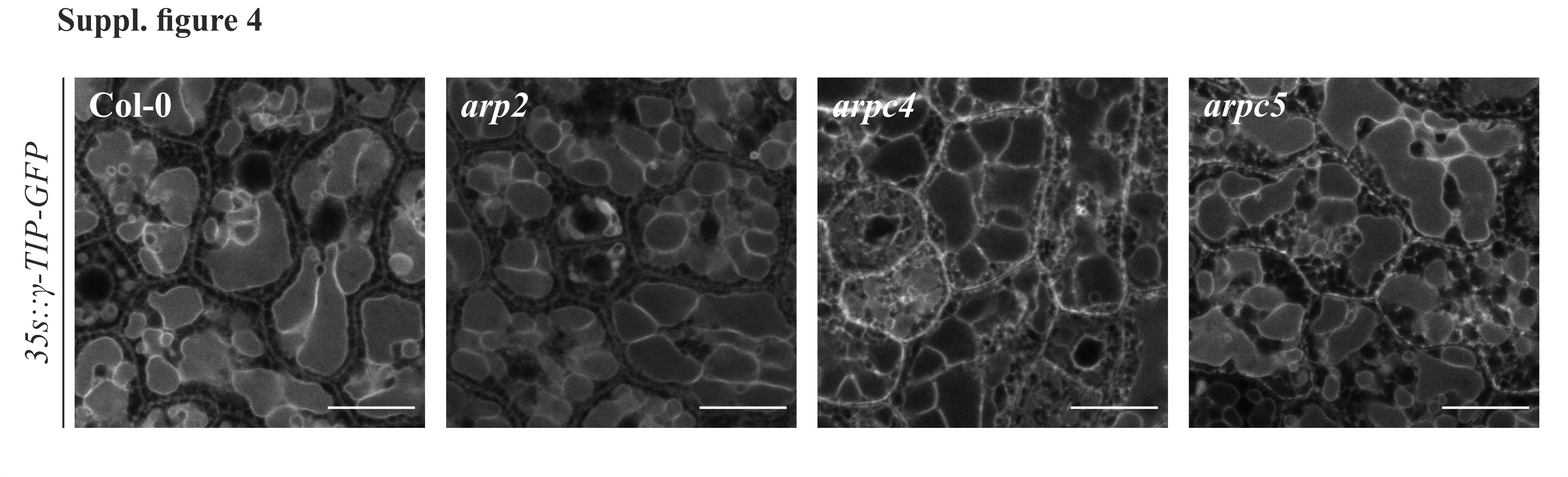

Supplement: FIGURE 4 — Vacuolar organization in wild-type and arp2, arpc4 and arpc5 in 1DAG cotyledon expressing the lytic vacuole γTIP-mCherry marker. Scale bar = 10 μm. [file Image_4.TIF]
